# Transgene stacking in potato using the GA*A*NTRY system

**DOI:** 10.1186/s13104-019-4493-8

**Published:** 2019-07-25

**Authors:** Kent F. McCue, Ethan Gardner, Ronald Chan, Roger Thilmony, James Thomson

**Affiliations:** Crop Improvement and Genetics, Western Regional Research Center, USDA-ARS, Albany, CA USA

**Keywords:** GA*A*NTRY, Gene-stacking, Site-specific recombinase, *Solanum tuberosum*

## Abstract

**Objective:**

GA*A*NTRY (Gene Assembly in *Agrobacterium* by Nucleic acid Transfer using Recombinase technologY) is a flexible and effective system for stably stacking multiple genes within an *Agrobacterium* virulence plasmid Transfer-DNA (T-DNA). We examined the ability of the GA*A*NTRY *Agrobacterium rhizogenes* ArPORT1 ‘10-stack’ strain to generate transgenic potato plants.

**Results:**

The 28.5 kilobase 10-stack T-DNA, was introduced into Lenape potato plants with a 32% transformation efficiency. Molecular and phenotypic characterization confirmed that six of the seven tested independent transgenic lines carried the entire desired construct, demonstrating that the GA*A*NTRY 10-stack strain can be used can be used in a tissue culture-based callus transformation method to efficiently generate transgenic potato plants. Analysis using droplet digital PCR showed that most of the characterized events carry one or two copies of the 10-stack transgenes and that ‘backbone’ DNA from outside of the T-DNA was absent in the transgenic plants. These results demonstrate that the GA*A*NTRY system efficiently generates high quality transgenic potato plants with a large construct of stacked transgenes.

**Electronic supplementary material:**

The online version of this article (10.1186/s13104-019-4493-8) contains supplementary material, which is available to authorized users.

## Introduction

*Agrobacterium* are soil microbes that have been harnessed for their ability to transfer DNA into plant cells [1, 2]. This technique has revolutionized agriculture, providing a way to identify and test gene functions and to transfer superior trait genes into crops without the added and often unwanted genes that come along during breeding schemes. An important aspect associated with the transfer of DNA into plants is the stability of the insertion event. *Agrobacterium* T-DNAs can sometimes be incomplete or concatenated repeats [3], exhibit genetic instability or gene silencing [4]. *Agrobacterium*-mediated transformation of plants with one or a few genes is relatively routine, but the assembly and transformation of large constructs carrying multiple genes and their efficient use to generate high-quality transgenic plants has been a challenge.

Previous research developed the GA*A*NTRY (Gene Assembly in *Agrobacterium* by Nucleic acid Transfer using Recombinase technologY) system for transgene stacking [5]. The system is based on the combined use of unidirectional integration and excision controlled by three site-specific serine recombinases and has been shown to be an effective and stable system for stacking multiple genes within an *Agrobacterium* virulence plasmid T-DNA [5, 6]. The gene stacking system utilizes easy-to-handle ‘P Donor’ and ‘B Donor’ cloning vectors for the insertion of sequences of interest. The P and B Donor vectors contain either *attP* or *attB* recognition sites enabling precise integration into the GA*A*NTRY ArPORT1 strain. The resulting *Agrobacterium* strain can then be directly used for plant transformation. The gene stacking strategy is efficient, precise, modular, and allows control over of the orientation and order in which genes are stacked within the T-DNA. The stacking process was previously demonstrated to successfully assemble a 28.5 kb 10-stack T-DNA construct containing ten cargo sequences, including eight transgenes that confer functional phenotypes in transgenic *Arabidopsis* plants [5]. The 10 stack T-DNA contains eight transcriptional units that confer functional phenotypes and one cargo (inserted twice) was the TBS [7] insulator sequence that blocks interactions between promoters and nearby enhancers. The transcriptional units included the *sul1* [8], firefly *luciferase* [9], *eGFP* [10], *bar* [11], *uidA* [12], *CsMybA* [13], *tdTomato* [14], and *nptII* [15] genes. The 10-stack construct was shown to produce high quality events that contain low copies of a complete T-DNA with rare incorporation of vector ‘backbone’ sequence in model plant *Arabidopsis* [5].

We wanted to examine the ability of the GA*A*NTRY ArPORT1 10-stack strain to produce stable clean single copy transgenic events in the important crop species of potato. Potato is a member of the *Solanaceae* family, which contains multiple other crop species and utilizes a tissue culture-based callus transformation method, which is distinctly different than the floral dip transformation method used for *Arabidopsis* [5].

We present the utilization of the GA*A*NTRY transgene stacking system to produce genetically engineered Lenape potato plants carrying the 28.5 kb ‘10-stack’ T-DNA. Multiple independent transgenic events were produced, phenotypically and molecularly characterized. Our analysis evaluated both the fidelity and completeness of T-DNA integration, and the copy number of the inserted sequences within the potato genome.

## Main text

### Results and discussion

**Fig. 1:**
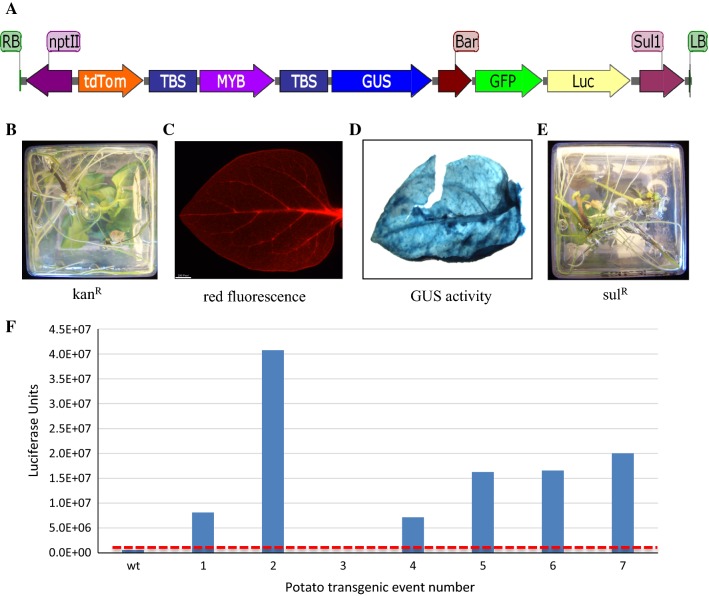
Diagram of the GA*A*NTRY 10-stack construct and phenotypes in transgenic potato plants. **a** Schematic representation of the GA*A*NTRY 10 stack T-DNA. **b** Transgenic potato shoots rooting in media containing 100 mg/L kanamycin (observed from the bottom of the magenta box). **c** Red fluorescence observed in a transgenic potato leaf. **d** Leaf GUS activity. **e** Transgenic potato shoots rooting in media containing 25 mg/L sulfadiazine. **f** Firefly luciferase activity measured in wildtype and seven 10-stack transgenic potato plants. The red dashed line marks the level of background luminescence detectable in wildtype potato leaf samples. Wildtype potato plants do not root on kanamycin or sulfadiazine media and do not exhibit detectable red fluorescence or GUS activity under these assay conditions

The GA*A*NTRY ArPORT1 10-stack strain was previously assembled and validated [5]. Details and the GenBank accessions for the donor plasmids carrying the promoter and gene sequences assembled within the 10-stack assembly are described in Additional file 2: Table S1. A simplified diagram of the 28.5 kb 10-stack T-DNA is shown in Fig. 1a.

The 10-stack GA*A*NTRY strain was used to transform potato B5141-6 (cv. Lenape) in two independent transformation experiments. A total of 86 internodes were co-cultivated with the ArPORT1 10-stack strain. A total of 28 shoots regenerated and rooted under kanamycin selection. Resistance to kanamycin is conferred by the *nptII* transgene, which is located adjacent to the right border (RB) of the T-DNA (Fig. 1a). Nodal cuttings from the rooted kanamycin resistant plants were excised and rooted on media containing the sulfadiazine selection agent (resistance is conferred by *sul1*, located adjacent to the Left Border (LB) of the T-DNA). Twenty-five plants survived and rooted in the presence of sulfadiazine, indicating the presence and expression of the *sul1* resistance gene in 89% of the transgenic plants that were recovered. The overall transformation rate based on kanamycin selection was 32.6% and it was 29.1% if both kanamycin and sulfadiazine resistance was required. Ten randomly selected kanamycin-resistant independent T_0_ transgenic events were transferred to soil and grown in the greenhouse. Seven of the plants successfully grew to maturity and were further characterized.

The selection marker and reporter gene phenotypes of these seven transgenic potato plants were further examined. All seven plants exhibited resistance to kanamycin in tissue culture media (Fig. 1b) and produced leaves with detectable red fluorescence (Fig. 1c). Six of the events (85%) were positive for β-glucuronidase histochemical staining of leaves (Fig. 1d) and these same six events exhibited resistance to sulfadiazine by successfully rooting on sulfadiazine containing tissue culture media (Fig. 1e), but line 3 was sensitive to sulfadiazine and did not root. Luciferase enzyme activity was detected in in leaf protein extracts from six of the events, but line 3 lacked detectable activity (Fig. 1f).

**Fig. 2:**
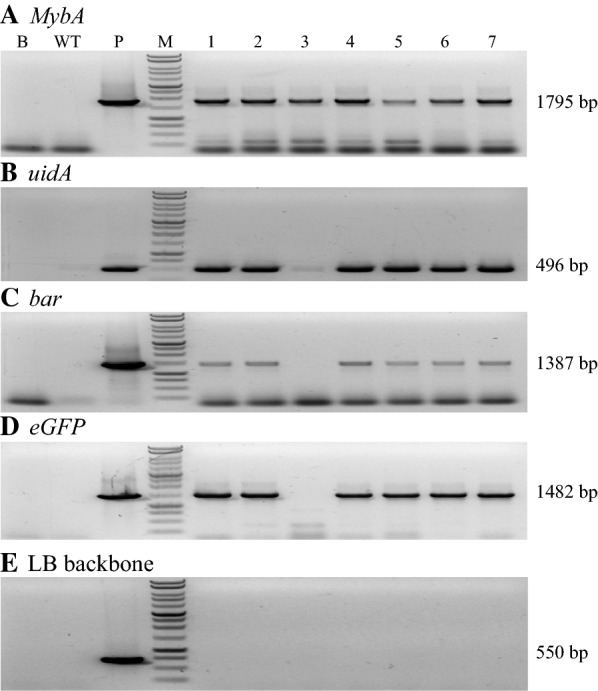
Genomic PCR screening of the 10-stack transgenic potato plants. Seven 10-stack potato transgenic events along with water (blank; B) and wildtype (WT) negative controls, and *A. rhizogenes* ArPORT1 10-stack genomic DNA (positive control; P) were analyzed for the presence of the *mybA* (**a**), *uidA* (**b**), *bar* (**c**), and *eGFP* genes (**d**). **e** The presence of LB ‘backbone’ sequence was also examined using primers that detect sequences outside of the T-DNA left border. The expected sizes each of the PCR amplicons are shown on the right of each panel

The presence of other portions of the 10-stack T-DNA was examined using PCR amplification of the transgene sequences using genomic DNA isolated from each transgenic event as template. The analysis illustrates that the *mybA* and *uidA* transgenes were detected in all of the seven tested lines, while the *bar* and *eGPF* transgenes were detected in six of the tested events, but not within line 3 (Fig. 2).

The *nptII* transgene copy number was measured for the seven selected transgenic events using droplet digital PCR [16]. Five of these events (71%) carried a single copy of the *nptII* transgene, while two events (29%) carried two copies (Additional file 1: Fig. S1). The transgene copy number for the *sul1* gene from these seven events was also determined, and the results show that four of the plants (57%) were single copy, two carried two copies (29%) and one event (line #3; 14%) lacked the *sul1* transgene (Additional file 1: Fig. S1).

The presence of sequences from outside of the T-DNA construct was also examined using genomic PCR screening. The analysis did not detect the presence of ‘backbone’ sequence from outside of the left border region in any of the seven transgenic lines, despite the fact that two of these lines contained two copies of *sul1* gene located adjacent to the Left Border of the T-DNA (Fig. 2e and Additional file 1: Fig. S1).

Taken together, these results suggest that four of the seven lines likely carried a single complete copy of the 28.5 kb 10-stack T-DNA construct. Two of the independent events appeared to carry two complete T-DNA copies, and a single event (line #3) contains only a partial T-DNA integration which lacks the *sul1*, *luciferase*, *eGFP,* and *bar* transgenes.

Together these results indicate that 4 out of 7 (57%) of the transgenic lines likely carry an intact, backbone free, single copy T-DNA integration demonstrating that the GA*A*NTRY system can be used in tissue culture-based transformation method for the production of transgenic potato with stacked gene constructs.

Rates of potato transformation for this study appeared to be somewhat lower than what we typically observe for potato transformation. This may be due to the larger size of the 10-stack T-DNA and/or characteristics of the ArPORT1 strain in potato transformation. Both the high rate of single copy transgenic production (57% of the tested lines) and the lower rates of transformation may be because the GA*A*NTRY T-DNA is launched directly from the low copy virulence plasmid, rather than a higher copy binary vector. The study by Oltmanns et al. [17] launched the T-DNA from the *picA* locus of the *Agrobacterium tumefaciens* chromosome and also recovered a lower transformation rate than observed for a binary vector. The lower transformation rate of the GA*A*NTRY system is not a significant problem, since multiple high-quality transgenic events were recovered within the small population of seven transgenic events that were analyzed.

Genetic engineering offers a powerful way to introduce or modify complex traits within crop plants. The GA*A*NTRY system enables the assembly and maintenance of multi-gene stacked constructs and can produce high quality transgenic events with low copy number transgene integrations that are free of sequences outside of the T-DNA in both Arabidopsis [5], and potato. Thus, the ArPORT1 10-stack GA*A*NTRY strain provides an effective technique to generate and efficiently introduce multiple genes into crop plants like potato and suggests that the GA*A*NTRY system will likely be useful in other *Solanaceous* plants as well.

### Methodology

Potato (*Solanum tuberosum*) B5141-6, the variety formerly known as Lenape [18] were maintained in a tissue culture chamber at 23 °C, 16 h light and grown in a greenhouse in Sunshine Mix #1 (Sungro Horticulture). Plant material was micropropagated in tissue culture on Shoot Media (Table 1) from excised 1 or 2 node segments (1 cm in size) when plantlets reached approximately 10 cm in height, or every 6–8 weeks.

**Table 1:** Potato culture media

Media^a^	SH	CC	Stage		
			I	II	III
MS^b^ with vitamins (g/L)	4.4	4.4	4.4	4.4	4.4
Sucrose (g/L)	30	20	20	20	30
pH^c^	5.6	5.4	5.6	5.6	5.6
GA_3_ (mg/L)	–	–	–	10	–
ZR^d^ (mg/L)	–	2	4	4	–
NAA^e^ (mg/L)	–	0.1	0.1	–	–
Kanamycin^f^ (mg/L)	–	–	100	100	100
Sulfadiazine^g^ (mg/L)	–	–	–	–	25
Carbenicillin^h^ (mg/L)	–	–	500	500	500

^a^SH: shoot maintenance; CC: co-cultivation; Stage I: callus induction; II: shoot induction; III: root induction

^b^MS: Murashige and Skoog [20] with Vitamins (M404, PhytoTechnology Laboratories). GA_3_: Gibberellic Acid 10 mg/ml stock in ethanol [0.29 mM]; (G745, Sigma-Aldrich) 0.29 μM final concentration

^c^The pH is adjusted to 5.4 or 5.6 with 0.1 N potassium hydroxide before addition of 0.2% Gelzan CM (G3251, PhytoTechnology Laboratories) for solidification

^d^ZR: Zeatin Riboside 1 mg/ml stock [4.56 mM] (Z875, PhytoTechnology Laboratories); final concentrations 9.1–18.2 μM

^e^NAA: 1-napthalene acetic acid (1 mg/ml stock, [5.4 mM] (N605, PhytoTechnology Laboratories); 0.54 μM final concentration

^f^Kanamycin 50 mg/ml stock; (K378, PhytoTechnology Laboratories) 100 μg/ml final concentration

^g^Sulfadiazine 25 mg/ml stock; 25 μg/ml final concentration

^h^Carbenicillin (C346, PhytoTechnology Laboratories) 500 μg/ml final concentration. Cefotaxime, timentin or vancomycin can be added to Stage I and II in addition to Carbenicillin as required for aggressive *Agrobacterium* strain

### *Agrobacterium rhizogenes* 10-stack strain

The *Agrobacterium rhizogenes* strain ArPORT1, harboring the 10-stack T-DNA contains the following 8 genes which confer functional phenotypes in plants: *sul1* [8] (sulfadiazine resistance), firefly *luciferase* [9] (luminescence), *eGFP* [10] (green fluorescence), *bar* [11] (herbicide resistance), *uidA* [12] (β-glucuronidase ‘GUS’ activity), *CsMybA* [13] (anthocyanin accumulation), *tdTomato* [14] (red fluorescence), and *nptII* [15] (kanamycin resistance). The promoters and terminators controlling transgene expression and the GenBank accessions for the donor vectors carrying these cargo sequences are shown in Additional file 2: Table S1 as previously described [5].

### Transformation and regeneration of transgenic potato plants

Potato transformation, selection and regeneration was conducted using a modified version of a previously described method [19]. Overnight *Agrobacterium* cultures were grown in a shaking incubator at 28 °C in Luria–Bertani (LB) medium containing 100 mg/l kanamycin. Cultures are washed twice and re-suspended in co-cultivation (CC) media (without Gelzan or hormones) to an OD_600_ of 0.2 AU. 1 cm long internode segments from 6 to 8 weeks old plantlets were excised and submerged in the *Agrobacterium* suspension and gently shaken for 20 min at 28 °C. Segments were rinsed with liquid CC media, blotted dry on sterile filter paper and placed on CC media for 2 days. Segments were transferred to Stage I media with weekly transfers. After 4 weeks segments were transferred to Stage II media with biweekly transfers. Shoots were transferred to Stage III media when they appeared, usually during weeks 4–6. Shoots that rooted in stage III were re-rooted in Stage III media to minimize non-transgenic escape plants. Rooted shoots were then transferred to soil, hardened off for 1–2 weeks and then planted in the greenhouse for the production of mini-tubers. See Table 1 for media recipes.

### Genomic DNA isolation and PCR

Genomic potato DNA was isolated using the Gentra PureGene DNA isolation kit (Qiagen). Droplet digital PCR was performed as previously described [16]. End-point PCR and droplet digital PCR amplification reactions were performed with the primers and probes shown in Additional file 3: Table S2.

### Detecting β-glucuronidase (GUS) activity

Lines were sampled and histochemically assayed for β-glucuronidase activity as previously described [12].

### Luciferase activity

Luminescence was analyzed using the Dual Luciferase Assay Kit (Promega). The manufacture’s protocol was followed, except a Luciferase Lysis Buffer (100 mM potassium phosphate; 1 mM EDTA; 10% glycerol; 1% Triton X; pH 7.8) was used for plant tissue extraction.

### Detection of red fluorescence

Red fluorescence was examined using a Leica MZ16FA dissecting microscope with a 590–650 nm bandpass filter. The excitation light provided was from 510 to 560 nm.

## Limitations

Transformation efficiency appeared lower than is typical for potato transformation with a binary vector containing a small (~ 8 kb) T-DNA. Whether this reduced transformation efficiency was due to the larger size of the 10-stack T-DNA or an attribute of the *Agrobacterium rhizogenes* ArPORT1 strain was not determined.

## Additional files

**Additional file 1: Fig. S1.** Transgene copy number measurements in seven potato 10-stack events.

**Additional file 2: Table S1.** 10-stack cargo sequences in Donor plasmids.

**Additional file 3: Table S2.** Primers and probes used for transgene detection.

## Data Availability

All data and material are available upon request. Mention of trade names or commercial products is solely for the purpose of providing specific information and does not imply recommendation or endorsement by the US Department of Agriculture. USDA is an equal opportunity provider and employer.

## Abbreviations

GA*A*NTRY: Gene Assembly in *Agrobacterium* by Nucleic acid Transfer using Recombinase technologY
ArPORT1: *Agrobacterium rhizogenes* strain that contains the genetic components required for GA*A*NTRY gene stacking within the virulence plasmid. Can be used directly for plant transformation
