# Supplementary material for: Transgene stacking in potato using the GAANTRY system
Source: BMC Res Notes. 2019 Jul 25;12:457. doi: 10.1186/s13104-019-4493-8 (PMC6659271; doi:10.1186/s13104-019-4493-8)
Supplement: Supplementary file 1 — Additional file 1: Fig. S1. Transgene copy number measurements in seven potato 10-stack events. [file 13104_2019_4493_MOESM1_ESM.pdf]

Fig S1
Transgene copy number measurements in seven potato 10-stack events.

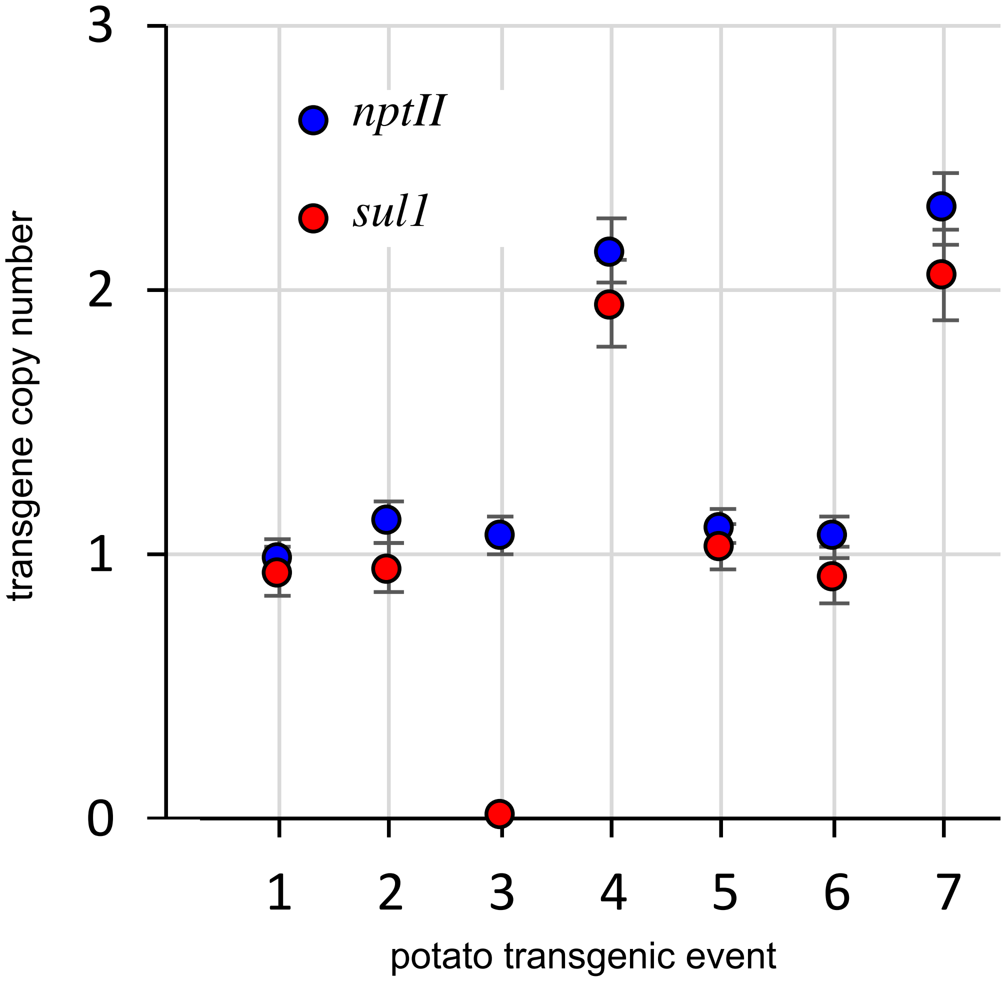

Fig S1. Transgene copy number measurements in seven potato 10-stack events. The *nptII* (blue) and *sul1* (red) transgene copy number was measured using ddPCR. The error values are Poisson 95% confidence intervals calculated by the QuantaSoft™ software.
