# Supplementary material for: Transgene stacking in potato using the GAANTRY system
Source: BMC Res Notes. 2019 Jul 25;12:457. doi: 10.1186/s13104-019-4493-8 (PMC6659271; doi:10.1186/s13104-019-4493-8)
Supplement: Supplementary file 2 — Additional file 2: Table S1. 10-stack cargo sequences in Donor plasmids. [file 13104_2019_4493_MOESM2_ESM.pdf]

Table S1
Ten-stack cargo sequences in donor plasmids.

Stack #	Transcriptional Unit/Cargo	Phenotype	Transcriptional Unit			TU Size (Kb)	GenBank Accession
			Promoter	CDS	Terminator		
1	sul1	sulfadiazine resistance	CaMV 35S	sul1, sulfonamide resistance 1	CaMV 35St	2.1	MG687276
2	Luc	luciferase activity	StUbi 409S	LUC, Firefly luciferase	nosT	4.0	MG687277
3	GFP	green florescence	StUbi7	eGFP, enhanced Green Florescence Protein	nosT	3.0	MG687278
4	bar	herbicide resistance	Nos	bar, bialaphos resistance	nosT	1.7	MG687279
5	GUS	β-glucuronidase acitivity	PrUbi	uidA, β-glucuronidase	nosT	4.4	MG687280
6	TBS	enhancer blocking insulator	N/A	N/A	N/A	2.2	MG687281
7	MYBA	anthocyanin accumulation	AtHTH	Citrus sinsensis MybA	nosT	3.4	MG687282
8	TBS	enhancer blocking insulator	N/A	N/A	N/A	2.2	MG687281
9	tdTom	red florescence	CaMV 35S	codon optimized tdTomatoER	nosT/35St	3.0	MG687283
10	nptII	kanamycin resistance	AtUbq10	nptII, neomycin phosphotransferase II	Ubq10t	2.3	MG687284

* N/A not applicable

Abbreviations: CaMV35S (double enhanced cauliflower mosaic virus 35S promoter); StUbi 409S (Solanum tuberosum ubiquitin 409S promoter); StUbi7 (Solanum tubersosum ubiquitin 7 promoter); Nos (nopaline synthase promoter); PrUbi1 (Pinus radiata ubiquitin 1 promoter); AtHTH (Arabidopsis thaliana HOTHEAD promoter); AtUbq10 (Arabidopsis thaliana ubiquitin 10 promoter); TBS (Petunia hybrida Transformation Booster Sequence); CaMV35St (cauliflower mosaic virus 35S 3' terminator) nosT (nopaline synthase 3' terminator); nosT/35St (nopaline synthase and CaMV35S double terminator); Ubq10t (Arabidopsis thaliana ubiquitin 10 3' terminator)
