# Supplementary material for: Transgene stacking in potato using the GAANTRY system
Source: BMC Res Notes. 2019 Jul 25;12:457. doi: 10.1186/s13104-019-4493-8 (PMC6659271; doi:10.1186/s13104-019-4493-8)
Supplement: Supplementary file 3 — Additional file 3: Table S2. Primers and probes used for transgene detection. [file 13104_2019_4493_MOESM3_ESM.pdf]

Table S2
Primers and probes used for transgene detection

Name	Sequence 5′-3′	Annealing temperature (°C)	Template	Amplicon size (bp)
**HTHp65 F60**	CAAAAGCCACATAAGAAGTTGATGCAGAAGC	60	mybA	1795
**MoroMybA R60**	CGATACTAGTCTTACTTTGCATTGAGAAGATCCC			
**GUSi F5**	GGAGTATTGCCAACGAACCG	55	uidA	496
**GUSi R6**	CGAGTGAAGATCCCTTTCTTG			
**NOSp ERV F59**	CAGTGATATCATACATGAGAATTAAGGGAGTCACGTTATGACC	60	bar	1387
**StUbi7pro 90**	GACCAAATCTTGTCATCCTCATCAACAAAACTAATG			
**StUbi7p 100 F60**	GGCTAAGATCCAGGATAAGGAAGGGATTCC	60	eGFP	1482
**409s pro 1400 R60**	GCAAATTAGGGTATAATACTCGTTGATAATCGCACTTTAATC			
**RTAC LB3 F61**	GGACTGATGGGCTGCCTGTATCG	60	LB backbone	550
**SulI830 F60**	CCAGAGACCGAGGGTTAGATCATGC			
**TO43 Inv-2 F**	CTCCCATTACACATTCCTCCC	55	StInv2	128
**TO447 Inv-2 R**	GGTTGTTGAGGATCGGAAAG			
**TO435 Inv-2 Probe**	TCTCCGGCATTTTCCTCTCCTCT		FAM Probe	
**AB099 nptII F**	CCGGCTACCTGCCCATTC	60	nptII	79
**AB100 nptII R**	CGACAAGACCGGCTTCCAT			
**AB101 nptII Probe**	AACATCGCATCGAGCGAGCACGT		HEX Probe	
**Sul1 F**	CTGTCCGATCAGATGCAC	54	sul1	139
**Sul1 R**	CCTCAGCAATATCGGGATAG			
**Sul1 Probe**	TTTCAATCGACAGCTTCCAACCGG		HEX Probe	
